# Supplementary material for: Asynchronous magnetic resonance elastography: Shear wave speed reconstruction using noise correlation of incoherent waves
Source: Magn Reson Med. 2022 Oct 27;89(3):990–1001. doi: 10.1002/mrm.29502 (PMC9792433; doi:10.1002/mrm.29502)
Supplement: Supplementary file 1 — DATA S1 MATLAB code used to generate the numerical simulations of Figure 1 [file MRM-89-990-s002.zip › k-Wave/helpfiles/scaleFig.html]

scaleFig :: Functions (k-Wave)


# scaleFig

Resize current figure window.

## Syntax

```
scaleFig(horizontal, vertical)
```

## Description

`scaleFig` resizes the current figure window by the specified horizontal and vertical multipliers. For example, to increase the width of the current figure by 50%, use the syntax `scaleFig(1.5, 1)`.

## Inputs

|  |  |
| --- | --- |
| `horizontal` | size multiplier for the horizontal direction |
| `vertical` | size multiplier for the vertical direction |

## See Also

`set`, `get`
